# Modelling attrition and nonparticipation in a longitudinal study of prostate cancer

**DOI:** 10.1186/s12874-018-0518-6

**Published:** 2018-06-20

**Authors:** Samantha Spiers, Evrim Oral, Elizabeth T. H. Fontham, Edward S. Peters, James L. Mohler, Jeannette T. Bensen, Christine S. Brennan

**Affiliations:** 1 LSUHSC, School of Public Health, Biostatistics Program, New Orleans, USA; 2 LSUHSC, School of Public Health, Epidemiology Program, New Orleans, USA; 3 Department of Urology, Roswell Park Cancer Institute, Buffalo, USA; 4 Lineberger Comprehensive Cancer Center, UNC-Chapel Hill, Chapel Hill, USA; 5 LSUHSC, School of Public Health, Health Policy and Systems Management Program, New Orleans, USA

**Keywords:** Unit nonresponse, Nonresponse bias, Attrition, Longitudinal study, Prostate cancer

## Abstract

**Background:**

Attrition occurs when a participant fails to respond to one or more study waves. The accumulation of attrition over several waves can lower the sample size and power and create a final sample that could differ in characteristics than those who drop out. The main reason to conduct a longitudinal study is to analyze repeated measures; research subjects who drop out cannot be replaced easily. Our group recently investigated factors affecting nonparticipation (refusal) in the first wave of a population-based study of prostate cancer. In this study we assess factors affecting attrition in the second wave of the same study. We compare factors affecting nonparticipation in the second wave to the ones affecting nonparticipation in the first wave.

**Methods:**

Information available on participants in the first wave was used to model attrition. Different sources of attrition were investigated separately. The overall and race-stratified factors affecting attrition were assessed. Kaplan-Meier survival curve estimates were calculated to assess the impact of follow-up time on participation.

**Results:**

High cancer aggressiveness was the main predictor of attrition due to death or frailty. Higher Charlson Comorbidity Index increased the odds of attrition due to death or frailty only in African Americans (AAs). Young age at diagnosis for AAs and low income for European Americans (EAs) were predictors for attrition due to lost to follow-up. High cancer aggressiveness for AAs, low income for EAs, and lower patient provider communication scores for EAs were predictors for attrition due to refusal. These predictors of nonparticipation were not the same as those in wave 1. For short follow-up time, the participation probability of EAs was higher than that of AAs.

**Conclusions:**

Predictors of attrition can vary depending on the attrition source. Examining overall attrition (combining all sources of attrition under one category) instead of distinguishing among its different sources should be avoided. The factors affecting attrition in one wave can be different in a later wave and should be studied separately.

## Background

Nonresponse occurs when a sampled subject fails to respond to a survey either partially (item nonresponse) or entirely (unit nonresponse). Unit nonresponse reduces sample size and study power. Significant differences between respondents and non-respondents can cause nonresponse bias, which is a type of selection bias [1].

Attrition occurs in a longitudinal study when a participant fails to respond to one or more study follow-up waves. A participant may skip one wave but subsequently respond to a later wave (intermittent) or may quit the study completely (drop-out). Since the accumulation of drop-outs over several waves can reduce the representativeness of the target population, nonresponse is even more of a concern in longitudinal studies. Problems from attrition in longitudinal studies are similar to those from nonresponse in cross-sectional surveys; they reduce study power and cause bias in estimates if certain subpopulations are over- or under-represented in the sample. Attrition can accumulate with each study wave, creating a final sample that could differ in characteristics from the original sample. Attrition due to death and decline in the health of study participants might cause particular problems in health-related studies of older people, because those who continue to participate would be healthier [2].

Nonresponse rates to epidemiologic studies have been observed to increase in recent years regardless of the disease studied, geographical region or age of the study population [3–10]. Morton et al. [11] abstracted information from 355 research articles and found that the declines in participation rates were particularly sharp in population-based case control studies, with an average decrease of 1.18% for cases and 1.49% for controls per year from 1970 to 2003. Such increases in nonresponse are paralleled in other disciplines. Curtin et al. [12] studied response to a telephone-based survey of consumer attitudes from 1979 through 2003 and observed a decrease in total response rate (from 72 to 48%) and an increase in refusal rate (from 19 to 27%) over time, with accelerating rates of decline in response for the period 1996–2003. In two longitudinal studies of recovery from coronary events, the drop-out rates ranged from 15 to 40%, causing a participant loss as high as 70% [13]. A longitudinal study of drug and alcohol use in adolescents lost almost 25% of the original cohort at 1 year follow-up [14].

Several theories exist for the increase in nonresponse. A decrease in social responsibility and an increase in privacy concerns have led individuals to be more reluctant to participate in surveys [15]. A rise in surveys has created research fatigue [8, 15]. Distrust of science and researchers, especially in non-European American (EA) communities, has hindered study recruitment efforts [16, 17]. Additional theories exist for increased attrition, such as loss of interest, moving and life changes [13]. Study fatigue also affects attrition, especially in older populations due to increased cognitive impairment and morbidity [13, 18]. Consequently, numerous studies have been conducted to determine which persons are more likely to be non-respondents or drop-outs. Males, people who are less educated, unemployed, not-married or of lower economic status are less likely to participate in cross-sectional studies. Results regarding age are less consistent, with some studies showing those who are younger having lower participation propensity than those who are older and other studies showing the opposite [8]. Generally, those who are more disadvantaged and in poorer health tend to be non-respondents [19]. Characteristics of drop-outs are similar. Males, people with less education, not-married, of lower economic status, of small household size, of poor health status, ethnic minority, or living in urban areas are at greater risk of attrition [13, 20, 21]. The effect of age on attrition is unclear, with some studies reporting increased attrition with old age and others reporting the opposite [22]. Longitudinal studies that focused on older cohorts generally reported a negative effect of old age on attrition. Older age, low education, and longer distance between the study center and the participant’s residence affected attrition in the Baltimore Longitudinal Study of Aging [23]. Increasing age and cognitive impairment were consistently related to increased attrition in a systematic review of 12 longitudinal studies in elderly populations [24].

Many strategies have been proposed to minimize nonresponse. Monetary incentives and advance letters have been shown to increase participation [15, 25]. Increasing interview attempts during evening hours might help to establish contact [1, 15]. Reminder letters can be sent to notify participants about when they will be contacted for the study. Interviewers’ interaction with potential participants might impact refusals; thus training interviewers regarding how to tailor their behavior while recruiting might help minimize refusals [1]. More experienced, extroverted and conscientious interviewers can increase participation [26]. Recruiting minority populations might require additional strategies. Organizing community outreach events, using racially diverse interviewers, and providing a toll free number might be beneficial in recruiting non-EA participants and for creating and maintaining trust [16, 27]. Note that an approach with a specific subgroup in a specific study may not work with a similar group under different circumstances. A combination of strategies should be used while monitoring the survey process in real time and modifying them as needed to decrease nonresponse and minimize racial/ethnic differences between respondents and non-respondents [28].

Attrition also can be minimized by offering incentives, sending postcards and scheduling telephone reminders. Collecting detailed contact information from participants at each wave could especially be useful in longitudinal studies with long follow-up times. A longitudinal study on adolescent drinking that maintained contact with 97% of its sample after 18 months initially asked participants not only for their personal contact information, but also for the contact information of adults and friends who could locate them if future contact was lost [29]. Re-contacting and re-interviewing participants who miss a study wave and bringing them back at later waves can help reduce overall attrition [30]. Newsletters may be used to update study participants on study progress in between waves to prevent attrition in longitudinal studies with long follow-up times. Community engagement, tracing noncontacts, utilizing mixed survey modes and providing incentives also have been shown to reduce attrition [21].

Statistical techniques can be used to adjust for nonresponse and attrition after collecting the data, but these procedures are not absolute. When auxiliary data exist, weighting procedures can reduce nonresponse bias, but they may bias estimates of standard errors. Small or large weights might create instabilities in estimates. Multiple imputation techniques can be used to replace missing data due to attrition by modeling the missingness; however, specifying the correct model from available data is difficult [31]. Optimal recruitment strategies should be implemented before and during data collection because post-survey adjustments are merely estimated remedies to the problems caused by nonresponse and attrition [28].

Current research on attrition usually does not differentiate between attrition through refusals and attrition through other reasons, such as death or lost to follow-up; however different sources of attrition have been shown to have different determinants [32]. In this study, we distinguished between different sources of attrition. We evaluated the potential predictors of attrition in the second wave of a study of prostate cancer (PCa). Research on attrition is commonly based on the characteristics of non-respondents at wave 1 to explain attrition in later waves, which ignores the role of events after wave 1. However, attrition models fit to early waves may become less predictive of attrition in future waves [32]. In order to compare the differences between the two waves of the same study group, we defined “nonparticipation” as “attrition through refusals”. We compared these findings with our previous nonparticipation analyses of the same Louisiana (LA) cohort [28] and evaluated if the factors affecting refusals were the same in each wave. Specifically, we assessed if racial differences regarding refusals were consistent over time.

## Methods

### The North Carolina-Louisiana prostate cancer project (*Wave 1*)

The North Carolina-Louisiana Prostate Cancer Project (PCaP) is a multidisciplinary population-based case-only study designed to identify racial and geographic influences on PCa aggressiveness. The study collected information on social, individual, and tumor level factors. Eligibility criteria included living in the North Carolina (NC) or LA study areas, having first diagnosis of histologically confirmed adenocarcinoma of the prostate, being 40–79 years old at PCa diagnosis, being able to complete the interview in English, living outside an institution, not being cognitively impaired or physically severely debilitated, and not being under the influence of severe medication or alcohol, or apparently psychotic at the time of the interview [33]. Demographic and socioeconomic characteristics of LA participants in PCaP (PCaP-LA) were described in Brennan et al. [34]. The PCaP-LA cohort started enrollment in September 2004 but suspended accrual in August 2005 due to Hurricane Katrina. This study phase is referred to as the pre-Katrina (Pre-K) sample, which enrolled 119 African-Americans (AAs) and 94 EAs. The post-Katrina (Post-K) enrollment resumed in September 2006 and completed in August 2009 with 506 AA and 508 EA participants [28, 34].

### The quality of life in prostate cancer project (*Wave 2*)

The Quality of Life in Prostate Cancer Project (Q-PCaP) is a follow-up study of the LA PCaP participants who were re-contacted 3–6 years after their initial interview. The follow-up study sought to investigate racial disparities in quality of life in men with PCa. All study participants enrolled in PCaP who completed the baseline interview and consented for future contact were eligible for Q-PCaP [34].

### Unit nonresponse in the PCaP-LA cohort

Our group previously collected auxiliary information on refusals of the PCaP-LA cohort by combining data from LA Tumor Registry (LTR), U.S. census tract and PCaP eligibility forms, and evaluated factors affecting nonparticipation in PCaP with a specific focus on race, PCa diagnosis age, and study phase (Pre-K vs Post-K) [28]. Results showed that older age for AAs (≥70 years), high neighborhood poverty for EAs, and study phase for both races were significant predictors of nonparticipation among eligible PCaP-LA research subjects [28]. In this study, we compared previous findings from wave 1 [28] with the current analyses from wave 2 to evaluate whether the factors affecting nonparticipation had changed with respect to the waves.

### Measures

Same or equivalent characteristics to those modeled previously [28] were included in our analyses to allow for comparisons; however in the current analyses, the aggregate and LTR based data used previously were replaced by individual-level data collected at wave 1. Age at diagnosis, race, and study phase were categorized as before [28]. We replaced the Gleason score and tumor stage used previously [28] by cancer aggressiveness, which is a composite score of Gleason score, tumor stage, and PSA at PCa diagnosis [35]. Census tract poverty was replaced with income and categorized as in Song et al. [35]. Rural density and parish were excluded in the current analyses since these factors pertained to Hurricane Katrina and thus to wave 1. We included additional factors in our models. Education was dichotomized as ≤high school and > high school as in Song et al. [35]. The Rapid Estimate of Adult Literacy in Medicine (REALM) short form [36], which measures health literacy, was categorized as ≤sixth grade (scores 0 to 44), seventh to eighth grade (scores 45 to 60), or high school (scores 61 to 66). Patient provider communication (PPC) score was measured using a 5-items indicator and adapted subscales from the Primary Care Assessment Survey 1995 Safran/The Health Institute [37], where higher scores indicate more positive communication between the patient and provider during PCa treatment. The Charlson Comorbidity Index (CCI) [38] was constructed from a comorbidity questionnaire where higher scores indicate more comorbidities.

### Statistical analyses

Binomial exact tests were used to compare sources of attrition in wave 2 with respect to race. Pearson chi-square tests and two-sample t-tests were used to assess associations between characteristics of wave 1 respondents and participation status in wave 2. Attrition was modeled using multinomial logistic regression. Racial differences in the attrition sources were assessed using race-stratified logistic regression and Firth’s penalized likelihood models; Firth’s logistic regression models were used to solve the problem of separation and reduce the bias of the maximum likelihood estimates due to low event rates after stratification. Survival analyses were performed to assess the impact of the follow-up time (time between the two waves) on participation. Kaplan-Meier estimates were calculated assuming that the event of interest is participation in wave 2. Time to event was considered to be the time since wave 1 (in years) and calculated as follows: For respondents of wave 2, the time difference between wave 1 and wave 2 interviews was calculated. Time to event was assigned a zero for the 46 participants who refused further contact at wave 1 interview. All drop-outs were considered to be censored observations, and the time to event was calculated as the time difference between wave 1 interview date and February 28, 2013, which was the last day of wave 2 data collection. To use the Kaplan Meier estimation technique, we assumed that the non-informative censoring assumption was satisfied by assuming time to participate in wave 2 (survival time) is independent of time to drop-out after wave 1 (censoring time). Additionally, we assumed that the participation probabilities were the same for research subjects recruited early and recruited late in PCaP. The Wilcoxon test was used to assess differences in participation probabilities between races. All statistical analyses were performed using SAS 9.4 (SAS Institute, Inc., Cary, North Carolina).

## Results

Of 1227 PCaP LA participants, 46 refused further contact at the time of wave 1. Contact was attempted for the remaining 1181 participants of whom 118 were deceased, 23 were too frail at the time of wave 2, 87 were lost to follow-up, and 189 refused to participate. The reasons for attrition stratified by race are shown in Table 1. The most common reason for attrition among AAs was active refusal, followed by lost to follow-up. In EAs, the most common reason for attrition also was active refusal, but followed by being deceased. More AAs than EAs dropped out overall (*p* = 0.001), were lost to follow-up (*p* < 0.001) and passively refused (interview was scheduled but never completed) to participate in wave 2 (*p* = 0.005).

**Table 1:** Sources of attrition in wave 2 overall and stratified by race^b^

Sources of Attrition	N (%)	AA^a^ (%)	EA^a^ (%)	*p*-value^c,d^
Deceased	118 (26)	59 (50)	59 (50)	1.000
Frail	23 (5)	13 (57)	10 (44)	0.532
Lost to Follow-Up	87 (19)	69 (79)	18 (21)	**< 0.001**
Passive Refusal	29 (6)	22 (76)	7 (24)	**0.005**
Active Refusal^e^	206 (45)	103 (50)	103 (50)	1.000
Total	463 (100)	266 (58)	197 (43)	**0.001**

^a^*AA* african american, *EA* european american

^b^Total participants of wave 2 = 764 men (62%)

^c^*p*-values were obtained from binomial exact tests separately for each reason to assess racial differences

^d^Significant *p*-values at Type I error 0.05 are bolded

^e^Includes 46 men who refused further contact at the time of wave 1

All baseline characteristics were significantly associated with attrition in wave 2 (Table 2). A larger percentage of wave 2 drop-outs were diagnosed between 60 and 69 years old, AA, had an income of $30,000 or less, had an education of high school or less, had a REALM score of high school, and had low cancer aggressiveness. While the average PPC score was lower among drop-outs than respondents (*p* = 0.011), the average CCI was higher (*p* = 0.030). Attrition rates also were calculated for each characteristic from the formulas given in the American Association of Public Opinion Research (AAPOR) [39] and provided in Table 2. For example, the attrition rate of men diagnosed between 40 and 59 years old was the number of drop-outs for that category, 142, divided by the total number of men for that category, 395, or 36%. The overall attrition rate was 38%; the greatest attrition rate occurred for wave 1 participants with high cancer aggressiveness (55%).

**Table 2:** Comparison of respondents and drop-outs in wave 2 by their characteristics^b^

Characteristic	Respondents (%)	Drop-Outs (%)	*p*-value^d, c^	Attrition Rate (%)
*Age at Diagnosis*			**0.009**	
40–59	33	31		36
60–69	45	40		35
70–79	22	29		45
*Race*			**< 0.001**	
AA^a^	47	58		43
EA^a^	53	43		33
*Study Phase*			**0.010**	
Post-K^a^	85	79		36
Pre-K^a^	15	21		46
*Income*			**< 0.001**	
$0–30,000	35	53		47
$30,001–70,000	34	32		36
≥ $70,001	32	16		23
*Education*			**< 0.001**	
≤ High School	42	61		47
> High School	58	39		29
*REALM* ^a^ *Score*			**< 0.001**	
≤ 6th Grade	19	34		52
7th or 8th Grade	21	20		37
High School	60	46		32
*Cancer Aggressiveness*			**< 0.001**	
Low	54	44		33
Intermediate	32	28		34
High	14	28		55
*PPC* ^a^ *Score*			**0.011**	
Mean (SD)	3.88 (0.79)	3.75 (0.89)		38
*CCI* ^a^			**0.030**	
Mean (SD)	0.97 (1.41)	1.15 (1.47)		38

^a^*AA* african american, *EA* european american, *Post-K* post-katrina, *Pre-K* pre-katrina, *REALM* rapid estimate of adult literacy in medicine, *PPC* patient provider communication, *CCI* charlson comorbidity index

^b^Missing data: Income (*n* = 155), Education (*n* = 2), REALM score (*n* = 2), Tumor aggressiveness (*n* = 83), PPC score (*n* = 16), CCI (*n* = 4)

^c^Pearson chi-square tests for categorical variables and two-sample t-tests for continuous variables were used to assess associations between characteristics and participation status. Note that for the PPC score, the *p*-value for equality of variances was 0.004, thus Satterhwaite’s approximation was used; for the CCI the *p*-value for equality of variances was 0.2878

^d^Significant *p*-values at Type I error 0.05 are bolded

Results of the multinomial logistic regression are shown in Table 3. Attrition because of death was less likely to occur among men who enrolled in wave 1 after Katrina than those who enrolled before it (OR = 0.6, 95% CI: 0.32–0.95). Men with high cancer aggressiveness at wave 1 were 4.5 times more likely to be deceased in wave 2 than those with low aggressiveness (95% CI: 2.54–7.86). The odds of attrition from death increased 1.3 times for every unit increase in CCI (95% CI: 1.12–1.46). Men who enrolled in wave 1 after Katrina were less likely to be drop-outs due to frailty (OR = 0.2, 95% CI: 0.08–0.67). Attrition due to lost to follow-up was less likely among men 60 and older at diagnosis than those younger than 60 (OR = 0.4, 95% CI: 0.22–0.74). AAs were 2.8 times more likely to be lost to follow-up than EAs (95% CI: 1.38–5.62). Men with an income $30,000 or less at wave 1 were 4 times more likely to drop out at wave 2 due to lost to follow-up than those with income more than $70,000 (95% CI: 1.59–10.41). Having an education of high school or less significantly increased the odds of being lost to follow-up compared to having a higher education (OR = 2.2, 95% CI: 1.10–4.23). Men with an income less than $70,000 were more likely to refuse to participate in wave 2 than those with higher income (OR = 1.8, 95% CI: 1.09–2.81). Men with a REALM score seventh to eighth grade at wave 1 were less likely to refuse to participate in wave 2 compared to those with a high school REALM score (OR = 0.6, 95% CI: 0.37–0.99). While high cancer aggressiveness at wave 1 increased the odds of refusal in wave 2 (OR = 1.6, 95% CI: 1.06–2.55), every one unit increase in PPC score decreased it 0.8 times (95% CI: 0.62–0.93).

**Table 3:** Estimated adjusted odds ratios for attrition with 95% Confidence Intervals^c, b^

			Attrition		
		Deceased	Frail	Lost to Follow-Up	Refusal
Age at Diagnosis	70–79	1.87 (0.99–3.51)	3.32 (0.94–11.73)	**0.37 (0.16–0.83)**	1.28 (0.81–2.03)
	60–69	1.00 (0.55–1.84)	0.62 (0.15–2.58)	**0.41 (0.22–0.74)**	0.93 (0.62–1.39)
Race	AA^a^	0.78 (0.46–1.34)	1.34 (0.42–4.26)	**2.79 (1.38–5.62)**	1.03 (0.71–1.51)
Study Phase	Post-K^a^	**0.55 (0.32–0.95)**	**0.24 (0.08–0.67)**	1.21 (0.58–2.52)	0.74 (0.48–1.16)
Income	$0–30,000	1.99 (0.91–4.35)	2.31 (0.38–13.94)	**4.07 (1.59–10.41)**	**1.78 (1.05–3.02)**
	$30,001–70,000	1.79 (0.85–3.74)	2.33 (0.43–12.67)	1.83 (0.70–4.79)	**1.75 (1.09–2.81)**
Education	≤ High School	1.30 (0.74–2.29)	0.76 (0.22–2.57)	**2.15 (1.10–4.23)**	1.35 (0.91–2.02)
REALM^a^ Score	≤ 6th	1.71 (0.85–3.44)	2.38 (0.52–10.84)	1.01 (0.48–2.15)	0.93 (0.56–1.56)
	7th - 8th	0.96 (0.50–1.86)	1.01 (0.24–4.31)	0.72 (0.33–1.55)	**0.61 (0.37–0.99)**
Cancer Aggressiveness	Intermediate	1.38 (0.78–2.44)	0.84 (0.29–2.41)	0.89 (0.47–1.68)	0.78 (0.52–1.15)
	High	**4.47 (2.54–7.86)**	0.28 (0.03–2.29)	1.77 (0.90–3.46)	**1.64 (1.06–2.55)**
PPC^a^ Score		0.80 (0.61–1.05)	0.79 (0.43–1.44)	1.10 (0.79–1.54)	**0.76 (0.62–0.93)**
CCI^a^		**1.28 (1.12–1.46)**	0.82 (0.53–1.26)	0.78 (0.61–1.01)	0.99 (0.88–1.12)

^a^*AA* african american, *Post-K* post-katrina, *REALM* rapid estimate of adult literacy in medicine, *PPC* patient provider communication, *CCI* charlson comorbidity index

^b^Referent categories: Age: 40–59 age group, Race: EA, Study phase: Pre-K, Income: ≥$70,001, Education: high school, REALM score: high school, and Cancer aggressiveness: low

^c^Significant *p*-values at Type I error 0.05 are bolded

The results from race-stratified logistic regression and Firth’s penalized likelihood models are presented in Table 4. The deceased and frail categories were combined due to the small numbers in the frail category. Both AAs and EAs who enrolled in wave 1 after Katrina were less likely to be deceased or frail than those who enrolled before Katrina (OR = 0.4, 95% CI: 0.20–0.88 and OR = 0.5, 95% CI: 0.22–0.94, respectively). Both AAs and EAs with high cancer aggressiveness were more likely to drop out because of death or frailty than those with low cancer aggressiveness (OR = 3.0, 95% CI: 1.34–6.62 and OR = 4.1, 95% CI: 1.94–8.63, respectively). Every one unit increase in CCI increased the odds of AAs being deceased or frail at wave 2 (OR = 1.3, 95% CI: 1.03–1.54), but the corresponding odds among EAs were not significant. AAs 60 and older at diagnosis were less likely to be lost to follow-up than AAs with younger age (OR = 0.4, 95% CI: 0.18–0.73). Although older age for EAs had similar associations with being lost to follow-up, these associations did not reach statistical significance. EAs with an income $30,000 or less were 8.7 times more likely to be lost to follow-up than those with an income more than $70,000 (95% CI: 1.88–39.77). Income was not a significant predictor of being lost to follow-up in AAs. Education was no longer a significant predictor for lost to follow-up for either AAs or EAs. AAs with high cancer aggressiveness were 2.5 times more likely to refuse to participate in wave 2 than those with low cancer aggressiveness (95% CI: 1.34–4.72). EAs with income $30,000 or less (OR = 2.8, 95% CI: 1.34–5.81) or with income between $30,001 and 70,000 (OR = 2.0, 95% CI: 1.07–3.58) were more likely to be refusals than those with income more than $70,000. Income was not a significant predictor of refusal in AAs. The odds of refusal in EAs decreased 0.7 times for every unit increase in PPC score (95% CI: 0.51–0.96). REALM score was not a significant predictor for refusal in either race.

**Table 4:** Estimated adjusted odds ratios for attrition in wave 2, stratified by race^f, g^

		Deceased or Frail^b, c^		Lost to Follow-Up^b, d^		Refusal^e^	
		AA^a^	EA^a^	AA	EA	AA	EA
Age at Diagnosis	70–79	2.03 (0.89–4.61)	2.30 (0.98–5.44)	**0.36 (0.14–0.93)**	0.27 (0.06–1.23)	1.03 (0.5–2.11)	1.38 (0.73–2.60)
	60–69	0.81 (0.37–1.73)	1.06 (0.45–2.48)	**0.37 (0.18–0.73)**	0.47 (0.15–1.53)	0.99 (0.57–1.73)	0.84 (0.46–1.53)
Study Phase	Post-K^a^	**0.41 (0.20–0.88)**	**0.46 (0.22–0.94)**	1.12 (0.48–2.62)	0.96 (0.25–3.72)	0.76 (0.40–1.45)	0.73 (0.38–1.40)
Income	$0–30,000	13.42 (0.84–214.98)	1.49 (0.58–3.78)	2.48 (0.86–7.13)	**8.65 (1.88–39.77)**	1.01 (0.44–2.30)	**2.79 (1.34–5.81)**
	$30,001–70,000	14.13 (0.88–225.87)	1.21 (0.56–2.63)	1.48 (0.49–4.45)	1.99 (0.44–9.05)	1.34 (0.61–2.95)	**1.96 (1.07–3.58)**
Education	≤ High School	1.37 (0.63–2.99)	1.15 (0.55–2.42)	1.92 (0.90–4.09)	2.18 (0.68–7.05)	1.34 (0.75–2.41)	1.29 (0.73–2.30)
REALM^a^ Score	≤ 6th	1.36 (0.58–3.21)	0.95 (0.26–3.44)	1.18 (0.51–2.74)	0.46 (0.07–2.91)	0.94 (0.47–1.86)	0.81 (0.33–2.00)
	7th - 8th	0.47 (0.18–1.20)	2.20 (0.98–4.92)	0.74 (0.30–1.80)	1.25 (0.35–4.52)	0.62 (0.31–1.21)	0.64 (0.30–1.39)
Cancer Aggressiveness	Intermediate	1.12 (0.55–2.30)	1.16 (0.55–2.45)	1.01 (0.49–2.06)	0.66 (0.20–2.23)	1.03 (0.59–1.80)	0.57 (0.32–1.01)
	High	**2.98 (1.34–6.62)**	**4.09 (1.94–8.63)**	2.17 (0.99–4.76)	1.17 (0.28–4.90)	**2.51 (1.34–4.72)**	1.07 (0.54–2.10)
PPC^a^ Score		0.77 (0.54–1.09)	0.91 (0.60–1.38)	1.23 (0.83–1.81)	1.05 (0.53–2.10)	0.76 (0.58–1.01)	**0.70 (0.51–0.96)**
CCI^a^		**1.26 (1.03–1.54)**	1.17 (0.98–1.39)	0.86 (0.65–1.12)	0.91 (0.61–1.37)	1.00 (0.83–1.21)	0.96 (0.80–1.15)

^a^*AA* african american, *EA* european american, *Post-K* post-katrina, *REALM* rapid estimate of adult literacy in medicine, *PPC* patient provider communication, *CCI* charlson comorbidity index

^b^Firth’s logistic regression models were used to reduce potential effects of low event rates

^c^The outcome variable was dichotomized as deceased or frail vs participant

^d^The outcome variable was dichotomized as lost to follow-up vs participant

^e^The outcome variable was dichotomized as refusal vs participant

^f^Significant *p*-values at Type I error 0.05 are bolded

^g^Referent categories: Age: 40–59 age group, Race: EA, Study phase: Pre-K, Income: ≥$70,001, Education: high school, REALM score: high school, and Cancer aggressiveness: low

**Fig. 1:**
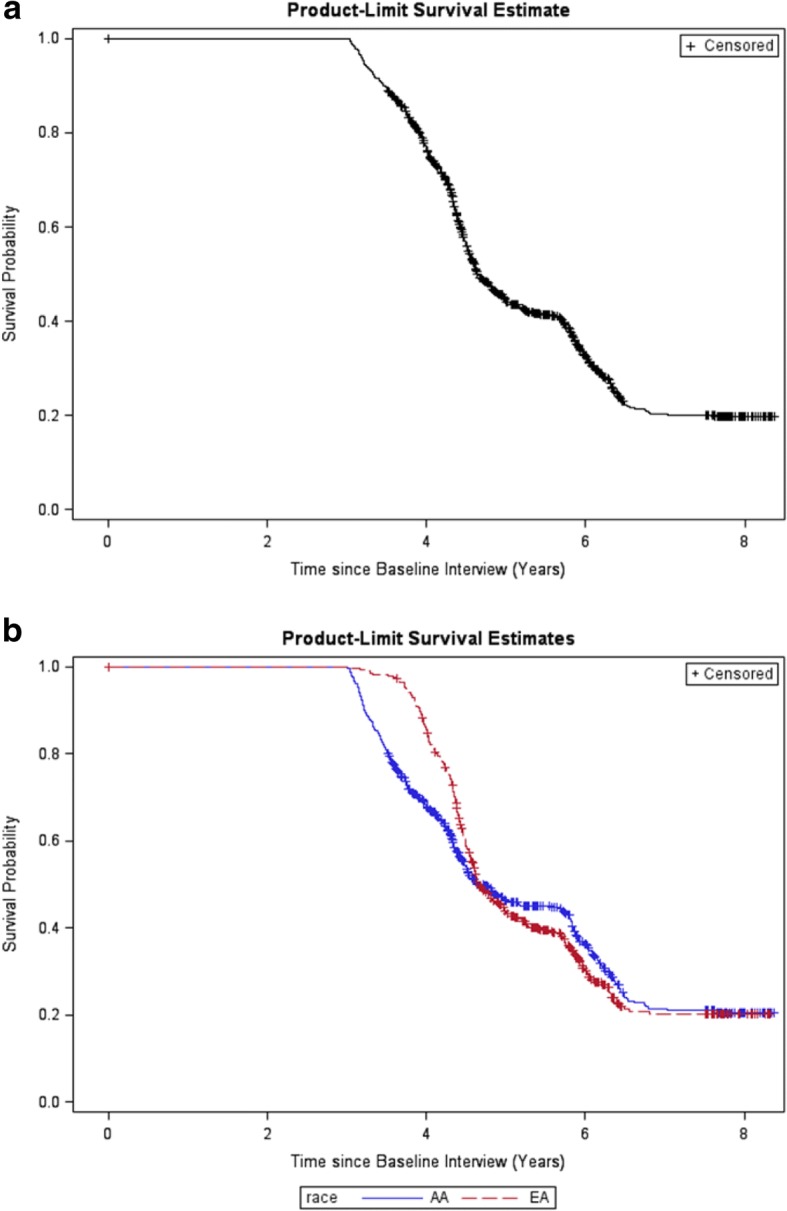
**a** Kaplan-Meier Survival Curves for wave 2 (overall).**b** Kaplan-Meier Survival Curves for wave 2 (stratified by race)

The overall and race-stratified product-limit estimates and 95% CIs, and their accompanying Kaplan-Meier survival curves are provided in Table 5 and Fig. 1, respectively. In the overall cohort, the probability of participating in wave 2 decreased to 50% (95% CI: 46.9–52.8), 4.64 years after the baseline interview. When stratified by race, the probability of participating in wave 2 decreased to 50% (95% CI: 45.6–54.1), 4.65 years after the baseline interview for AAs, and it decreased to 50% (95% CI: 45.7–53.8), 4.66 years after the baseline interview for EAs. The estimated curve for EAs was above the one for AAs until the curves crossed at 4.66 years after wave 1 (Fig. 1b). Although the probability of participating at wave 2 was higher for EAs initially, the probability of participating in wave 2 became slightly higher for AAs after 4.66 years. The probability of participating in wave 2 decreased to 20% both in the overall sample and in both races 8 years after the baseline interview. The Wilcoxon test for equality of participation probabilities was significant (*p* < 0.0001), which indicated a short-term difference in participation probabilities between races with respect to follow-up times.

**Table 5:** Kaplan-Meier survival curve estimates and 95% CIs at different time points, overall and stratified by race

	Product-Limit Estimates of Participation in Wave 2		
Time Since Wave 1 (Years)	Overall	AA^a^	EA^a^
3.00	1.0	1.0	1.0
4.00	0.766 (0.740–0.789)	0.682 (0.642–0.718)	0.851 (0.819–0.877)
4.64	0.500 (0.469–0.528)	0.502 (0.458–0.544)	0.507 (0.466–0.547)
4.65	0.497 (0.467–0.526)	0.500 (0.456–0.541)	0.506 (0.464–0.545)
4.66	0.493 (0.463–0.522)	0.500 (0.456–0.541)	0.500 (0.457–0.538)
4.70	0.489 (0.459–0.519)	0.499 (0.453–0.539)	0.491 (0.450–0.531)
5.00	0.441 (0.411–0.470)	0.461 (0.417–0.504)	0.434 (0.393–0.474)
6.00	0.325 (0.294–0.356)	0.363 (0.316–0.409)	0.301 (0.261–0.343)
7.00	0.203 (0.173–0.236)	0.214 (0.169–0.262)	0.203 (0.162–0.248)
8.00	0.198 (0.167–0.230)	0.204 (0.160–0.252)	0.203 (0.162–0.248)

^a^*AA* african american, *EA* european american

## Discussion

The results showed that enrollment in wave 1 before Katrina was a significant predictor of attrition due to death or frailty, both for the overall sample and for both races. High cancer aggressiveness significantly increased the odds of attrition due to death or frailty for both races, yet higher CCI increased the odds of attrition due to death or frailty only in AAs. The results for attrition due to lost to follow-up in the overall model were consistent with literature that has found minorities and those with lower education are more likely to be lost to follow-up [40]. Although lower education was significantly associated with being lost to follow-up in the overall model, the association did not reach significance when the model was stratified by race. For AAs, being younger than 60 at diagnosis was the only significant predictor for attrition due to lost to follow-up. After hurricane Katrina, the loss of rental housing inflated rental housing costs in the New Orleans area [41]. Younger AAs may have relocated to more affordable housing. For EAs, the only significant predictor for attrition due to lost to follow-up was low income (≤$30,000). EAs with low income may have been residing in rentals and had to relocate to more affordable rental units after Katrina. Also, homeowners relying on federal disaster assistance received payments several years after Katrina that often were not enough to rebuild their homes [41]; EAs of low income may have had a temporary residence during wave 1 and became lost to follow-up once they were able to rebuild or relocate.

For the same cohort, our group previously showed that older diagnosis age for AAs (≥70 years), low neighborhood poverty for EAs (< 20% of the households within the tract living in poverty), and study phase with respect to Katrina for both races were significant predictors of nonparticipation (refusal) in wave 1 [28]. Age and study phase were no longer significant predictors of nonparticipation in wave 2 for either race. Instead, high cancer aggressiveness for AAs, low income for EAs (≤ $70,000), and lower PPC scores for EAs were the significant predictors of nonparticipation in wave 2. One possible explanation for the opposite effect of income in EA nonparticipation might be that the compensation for participation in wave 1 was a maximum of $75, while it was only $25 in wave 2. This incentive decrease might have caused EAs with low income to be less interested in participating in wave 2. Another explanation can be provided based on the leverage-salience theory [42]: the salience and/or leverage of the survey features, such as busyness, monetary incentive amount, or willingness to contribute to PCa research, might have changed for EAs after wave 1. There may have been an additional survey feature that was not present at wave 1, such as experience with the interviewer in the first wave, which added to their leverage and changed their participation. Income did not alter AAs’ participation in either wave, which highlights the need for using different approaches to boost participation of AAs in PCa studies in addition to providing monetary incentives (see [28]). PPC score, which is associated with higher patient satisfaction [43, 44], has not been studied to our knowledge in nonresponse/attrition research. These results indicated that a more positive perception of PPC significantly decreased the odds of refusals, both in the overall sample and in EAs. PCa researchers should encourage providers to promote their patients’ ongoing study involvement throughout longitudinal data collection.

In longitudinal studies, longer follow-up periods are well known to be associated with higher attrition, but the degree of this association has not been studied well [45, 46]. The results from survival analyses showed that the participation probability of EAs was higher than the participation probability of AAs when the length of follow-up was shorter than 4.66 years. However, these probabilities were reversed when the follow-up time increased. Thus, racial differences may need to be considered when planning follow-up times. PCa researchers should keep the times between waves short when conducting longitudinal studies. However, the survival analyses were limited by lack of information on exact time of attrition. Further research needs to be done on the effect of exact time between waves on attrition to confirm these findings.

## Conclusions

We assessed factors affecting attrition in the second wave of a population-based study of PCa. Studies have been conducted in various populations, including elderly, to determine the effects of health on nonresponse and attrition [2, 13, 30]. But, to our knowledge, no such study exists specifically for PCa populations, which are prone to both aging and frailty. The large and approximately equal sample sizes of AAs and EAs enabled us to assess the racial differences in attrition. Our results verified the need for studying sources of attrition separately when possible; examining attrition without distinguishing between its different sources can cause separate factors to be missed. Our results also demonstrated the danger of using one wave of a longitudinal study to evaluate nonresponse in later waves. Unless the interval between waves is very short, strategies used to decrease attrition at an earlier wave may not be useful at the time of a subsequent wave because the salience and/or leverage of the study features might change for participants over time. The factors affecting attrition and nonparticipation should be studied constantly at each wave to tailor ongoing retention efforts in longitudinal studies.

## Abbreviations

AA: African american
AAPOR: The american association of public opinion research
CCI: Charlson comorbidity index
EA: European american
LA: Louisiana
LTR: Louisiana tumor registry
NC: North carolina
PCa: Prostate cancer
PCaP: The north carolina-louisiana prostate cancer project
Post-K: Post-katrina
PPC: Patient provider communication
Pre-K: Pre-katrina
Q-PCaP: The quality of life in prostate cancer project
REALM: The rapid estimate of adult literacy in medicine
